# Supplementary material for: Overexpression of the Rieske FeS protein of the Cytochrome b6f complex increases C4 photosynthesis in Setaria viridis
Source: Commun Biol. 2019 Aug 16;2:314. doi: 10.1038/s42003-019-0561-9 (PMC6697696; doi:10.1038/s42003-019-0561-9)
Supplement: Supplementary file 1 — Supplementary figures [file 42003_2019_561_MOESM1_ESM.docx]

**Supplementary figures**


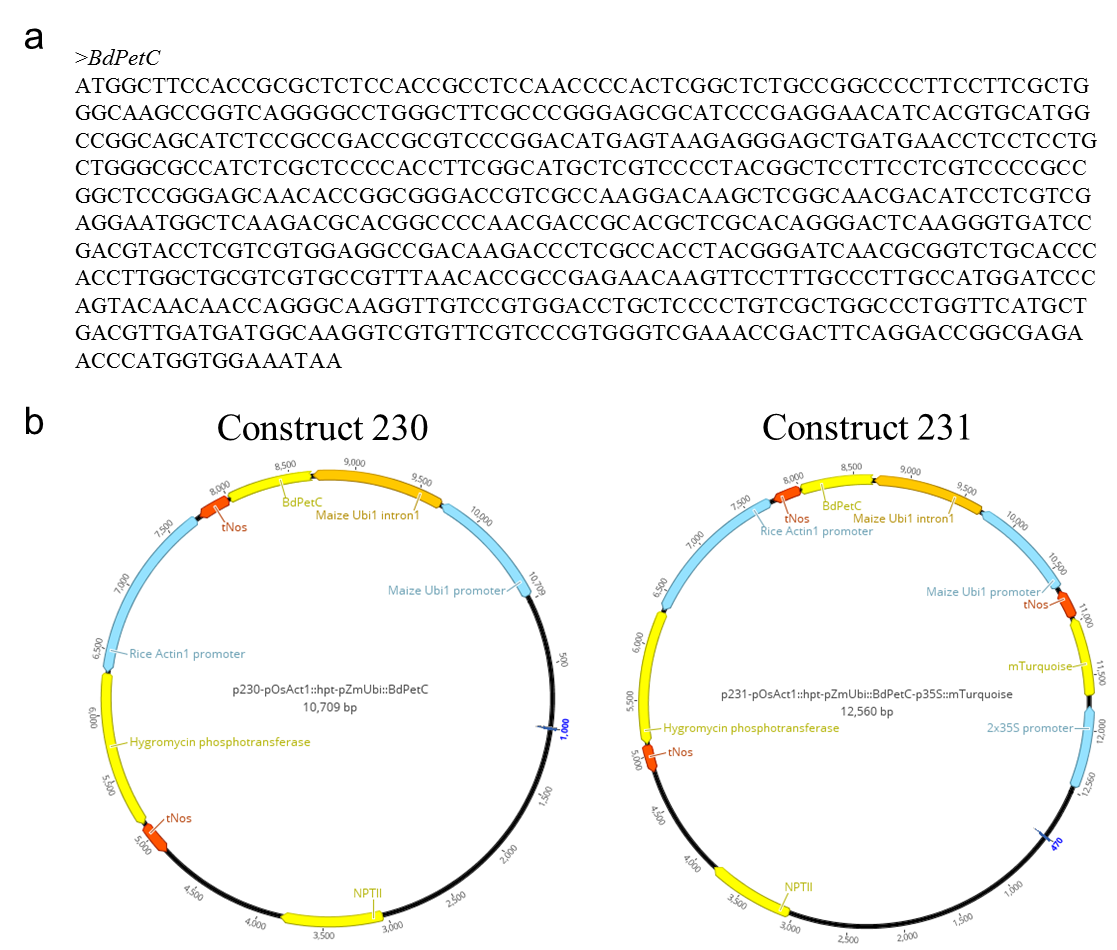


**Fig. 1**. Constructs used for Rieske FeS overexpression in *S.viridis* **a**. Coding sequence of the *PetC* gene from *B.distachyon* (*BdPetC*) domesticated for the Golden gate cloning system. **b**. Constructs for Rieske FeS overexpression containing the hygromycin phosphotransferase gene driven by the rice actin promoter and *BdPetC* gene driven by the maize ubiquitin promoter. Construct 231 contained an additional expression module for mTurquoise fluorescent protein under control of the 2x35S promoter. The bacterial terminator tNos was used in all transcription modules.

*
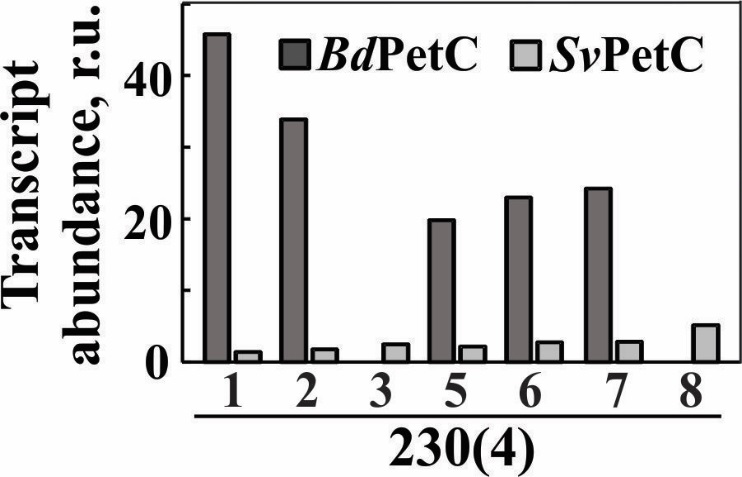
*

**Fig. 2**. Transcript abundance of the *PetC* gene from *B.distachyon* (*BdPetC*) or from *S.viridis* (*SvPetC*) analysed in T_1_ plants of the line 230(4) relative to the expression level of the reference genes for Ubiquitin, Elongation factor 1a and Beta tubulin.


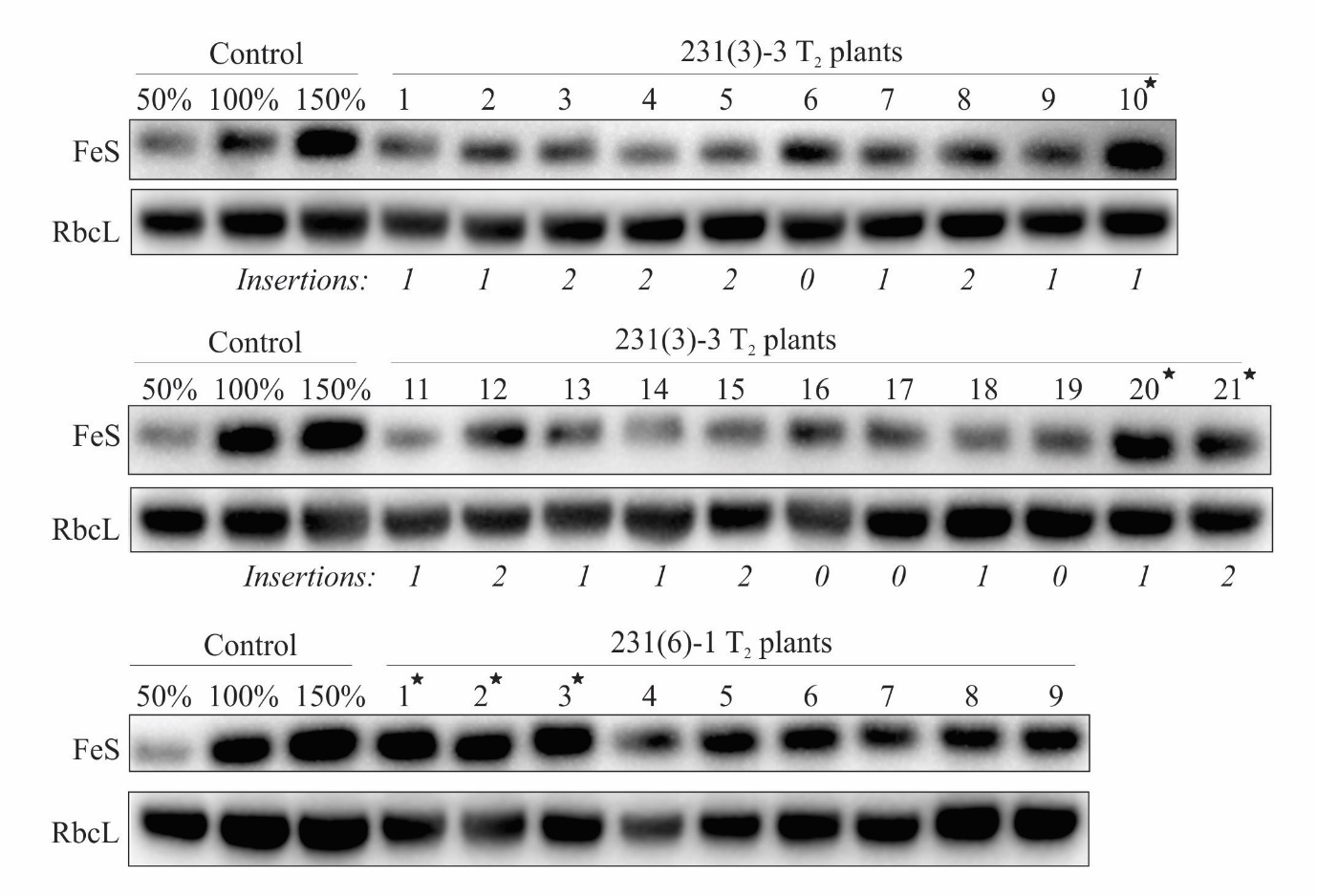


**Fig. 3**. Immunodetection of Rieske FeS and Rubisco large subunit (RbcL) in T_2_ plants of the lines 231(3)-3 and 231(6)-1 on leaf area basis. Insertion numbers indicate copy numbers of the hygromycin phosphotransferase gene; all T_2_ plants of the line 231(6)-1 had 4 insertions. Asterisks indicate plants used for gas-exchange analysis on Fig. 3.


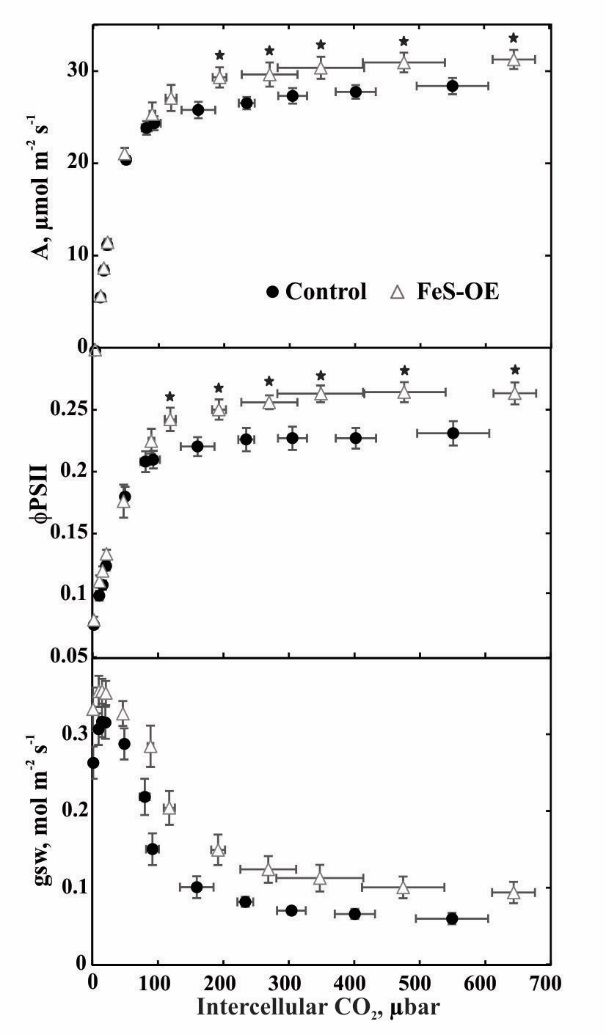


**Fig. 4**. CO_2_ response of CO_2_ assimilation rate (A), quantum yield of Photosystem II (φPSII) and stomatal conductance (gsw) in Rieske FeS overexpression T_2_ plants of the line 231(3)-3 (FeS-OE) and control plants measured at 1500 µmol m^-2^ s^-1^. Mean ± SE, n=3. Asterisks indicate statistically significant differences between transgenic and control plants (*P*<0.05).

**
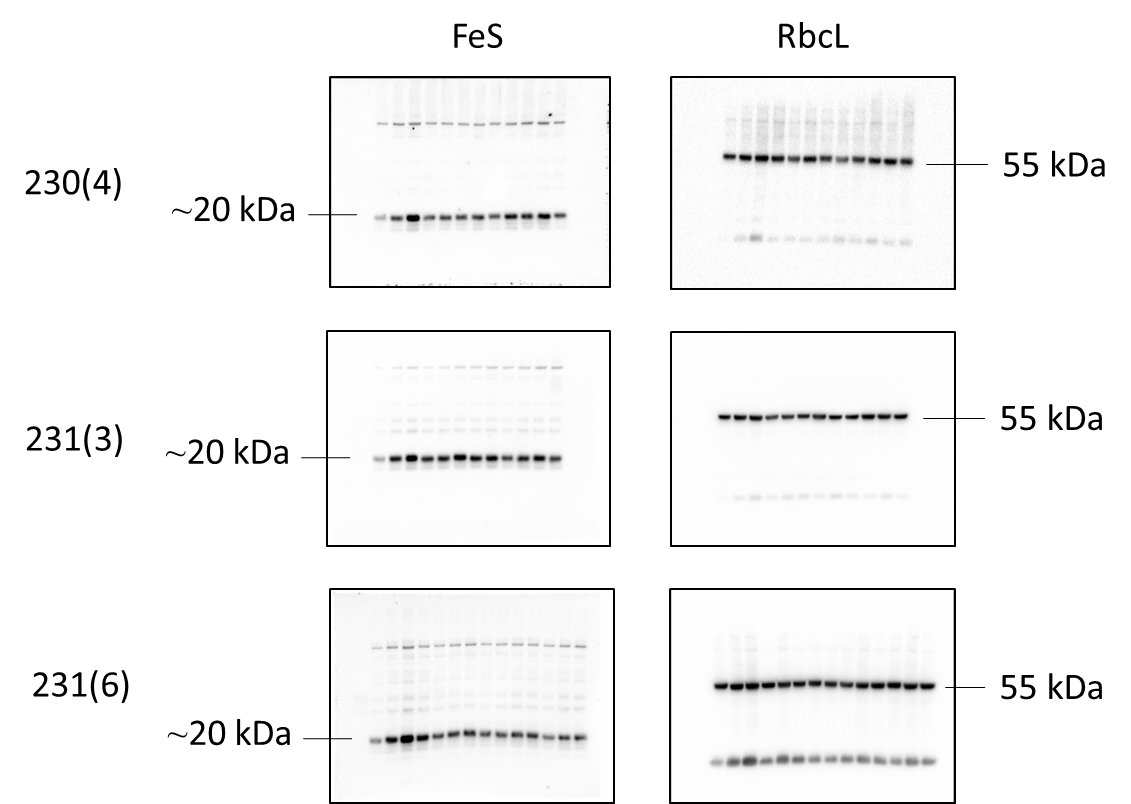
**

**Fig. 5.** Uncropped images of the membranes used for immunodetection shown in Fig. 1b.

**
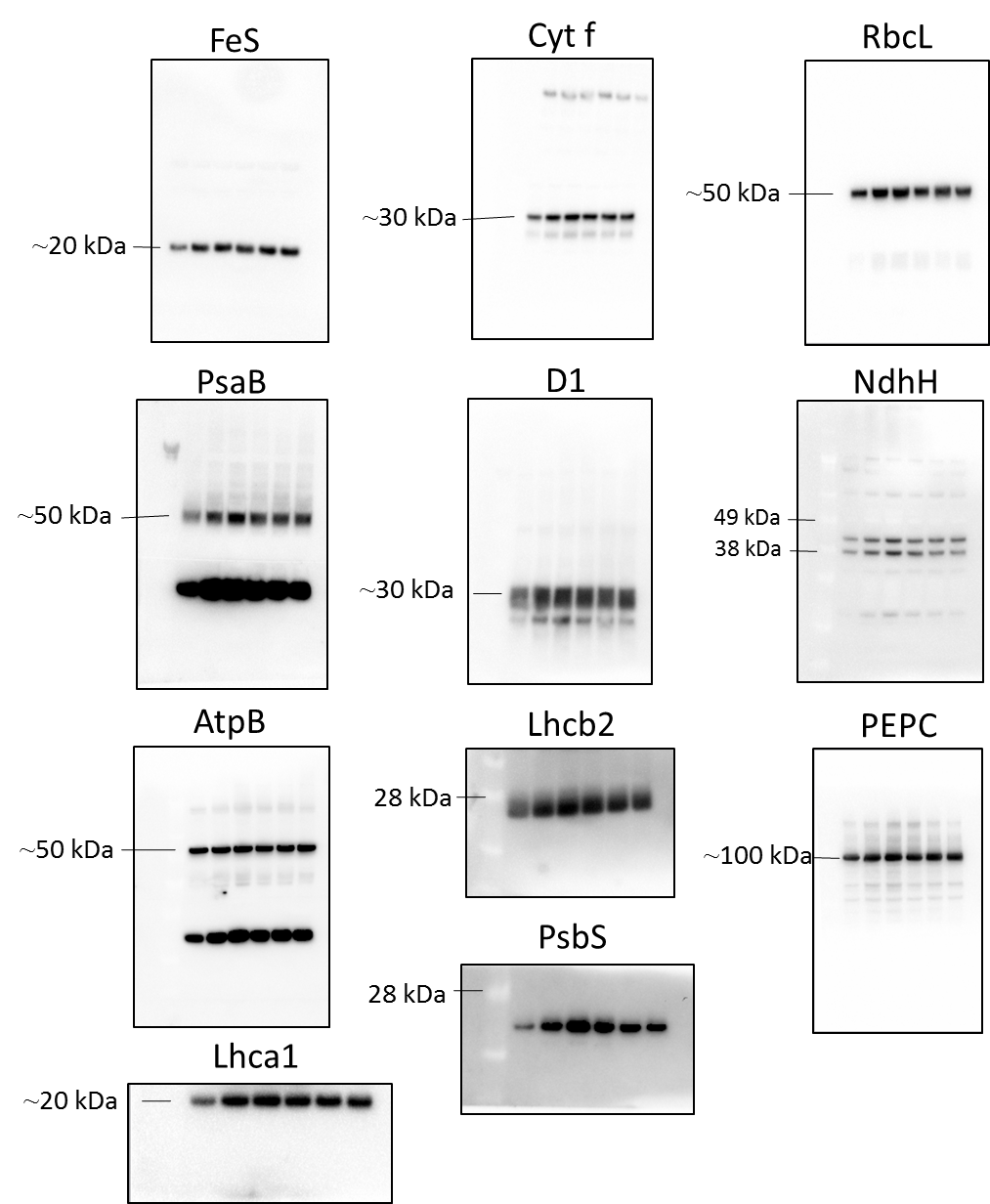
**

**Fig. 6.** Uncropped images of the membranes used for immunodetection shown in Fig. 2a.


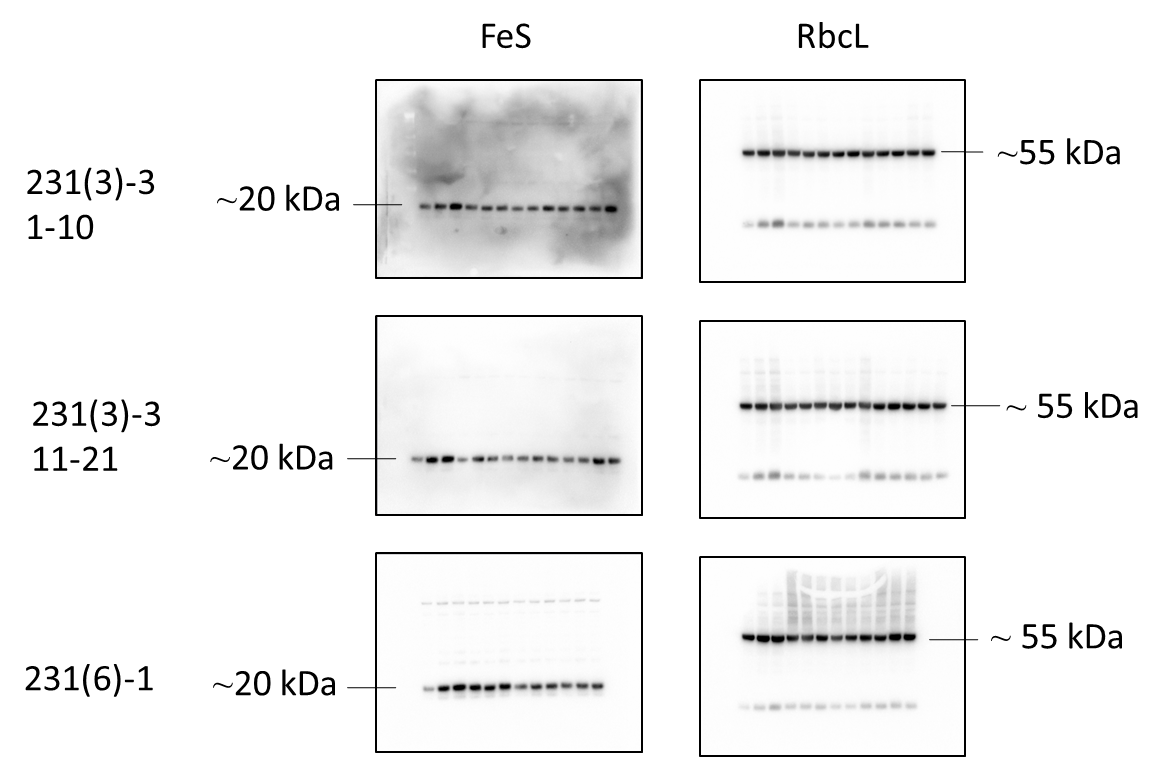


**Fig. 7.** Uncropped images of the membranes used for immunodetection shown in Supplementary Fig. 3.
