# Supplementary material for: Overexpression of the Rieske FeS protein of the Cytochrome b6f complex increases C4 photosynthesis in Setaria viridis
Source: Commun Biol. 2019 Aug 16;2:314. doi: 10.1038/s42003-019-0561-9 (PMC6697696; doi:10.1038/s42003-019-0561-9)
Supplement: Supplementary file 2 — Description of Additional Supplementary Files [file 42003_2019_561_MOESM2_ESM.docx]

**Description of additional supplementary files**

**Supplementary Data 1.** Data used for protein abundance displayed in figure 1c and figure 2b.

**Supplementary Data 2.** Data used for the graphs shown in figure 3.

**Supplementary Data 3.** Data used for the graphs shown in figure 4.

**Supplementary Data 4.** Data used for the graphs shown in figure 5.

**Supplementary Data 5.** Data used for the graphs shown in figure 6.
