# Supplementary material for: Overexpression of the Rieske FeS protein of the Cytochrome b6f complex increases C4 photosynthesis in Setaria viridis
Source: Commun Biol. 2019 Aug 16;2:314. doi: 10.1038/s42003-019-0561-9 (PMC6697696; doi:10.1038/s42003-019-0561-9)
Supplement: Supplementary file 8 — Reporting Summary [file 42003_2019_561_MOESM8_ESM.pdf]

nature research

Corresponding author(s): Maria Ermakova

Last updated by author(s): Jul 9, 2019

Reporting Summary

Nature Research wishes to improve the reproducibility of the work that we publish. This form provides structure for consistency and transparency in reporting. For further information on Nature Research policies, see Authors & Referees and the Editorial Policy Checklist.

Statistics

For all statistical analyses, confirm that the following items are present in the figure legend, table legend, main text, or Methods section.

n/a	Confirmed	
☐	☒	The exact sample size (*n*) for each experimental group/condition, given as a discrete number and unit of measurement
☐	☒	A statement on whether measurements were taken from distinct samples or whether the same sample was measured repeatedly
☐	☒	The statistical test(s) used AND whether they are one- or two-sided<br>*Only common tests should be described solely by name; describe more complex techniques in the Methods section.*
☒	☐	A description of all covariates tested
☒	☐	A description of any assumptions or corrections, such as tests of normality and adjustment for multiple comparisons
☐	☒	A full description of the statistical parameters including central tendency (e.g. means) or other basic estimates (e.g. regression coefficient) AND variation (e.g. standard deviation) or associated estimates of uncertainty (e.g. confidence intervals)
☐	☒	For null hypothesis testing, the test statistic (e.g. *F*, *t*, *r*) with confidence intervals, effect sizes, degrees of freedom and *P* value noted<br>*Give P values as exact values whenever suitable.*
☒	☐	For Bayesian analysis, information on the choice of priors and Markov chain Monte Carlo settings
☒	☐	For hierarchical and complex designs, identification of the appropriate level for tests and full reporting of outcomes
☒	☐	Estimates of effect sizes (e.g. Cohen's *d*, Pearson's *r*), indicating how they were calculated

Our web collection on statistics for biologists contains articles on many of the points above.

Software and code

Policy information about availability of computer code

Data collection	DualPAM V1.19, LI-6800 v 1.3.17, MultispeQ v 1.22
Data analysis	PhotosynQ web application (https://photosynq.org), Image Lab software (Biorad, Hercules, CA), Primer3 in Geneious R9.1.1 (https://www.geneious.com).

For manuscripts utilizing custom algorithms or software that are central to the research but not yet described in published literature, software must be made available to editors/reviewers. We strongly encourage code deposition in a community repository (e.g. GitHub). See the Nature Research guidelines for submitting code & software for further information.

Data

Policy information about availability of data

All manuscripts must include a data availability statement. This statement should provide the following information, where applicable:

- Accession codes, unique identifiers, or web links for publicly available datasets
- A list of figures that have associated raw data
- A description of any restrictions on data availability

The datasets analysed in this paper are included in this published article and supplementary information files. MultipeQ data are available at PhotosynQ web application (https://photosynq.org), project ID 3400. Plasmids used for generation of plants with Rieske FeS overexpression can be obtained from Addgene (deposit 77017). Further datasets generated during the current study as well as seeds of the WT and FeS-OE S.viridis are available from the corresponding author on request.

Field-specific reporting

Please select the one below that is the best fit for your research. If you are not sure, read the appropriate sections before making your selection.

☒ Life sciences ☐ Behavioural & social sciences ☐ Ecological, evolutionary & environmental sciences

For a reference copy of the document with all sections, see nature.com/documents/nr-reporting-summary-flat.pdf

Life sciences study design

All studies must disclose on these points even when the disclosure is negative.

Sample size	Eight plants were grown and analysed for each of 3 transgenic lines in T1 generation. Up to 30 plants were grown for each of 3 transgenic lines in T2 generation and plants with Rieske FeS overexpression confirmed by immunoblotting comprised 10-20% depending on the line. 3-4 T2 plants with the highest over-expression levels were used for analyses in each line.
Data exclusions	For MultispeQ data, measurements collected at significantly different light intensities were excluded, otherwise no data were excluded from the analysis.
Replication	Experiments were partially replicated in T1 and T2 generations for 3 independent lines.
Randomization	Plants of different genotypes were placed randomly in growth chambers to reduce any position effects.
Blinding	For T1 generation, gas-exchange and protein analyses for all plants were performed independently and correlated afterwards.

Reporting for specific materials, systems and methods

We require information from authors about some types of materials, experimental systems and methods used in many studies. Here, indicate whether each material, system or method listed is relevant to your study. If you are not sure if a list item applies to your research, read the appropriate section before selecting a response.

Materials & experimental systems

n/a	Involved in the study	
☐	☒	Antibodies
☒	☐	Eukaryotic cell lines
☒	☐	Palaeontology
☒	☐	Animals and other organisms
☒	☐	Human research participants
☒	☐	Clinical data

Methods

n/a	Involved in the study	
☒	☐	ChIP-seq
☒	☐	Flow cytometry
☒	☐	MRI-based neuroimaging

Antibodies

Antibodies used	PetC (Agrisera, AS08 330), PsaB (Agrisera, AS10 695), D1 (Agrisera, AS10 704), NdhH (Agrisera, AS16 4065), AtpB (Agrisera, AS05 085), Lhca1 (Agrisera, AS01 005), Lhcb2 (Agrisera, AS01 003), PsbS (Agrisera, AS09 533), PEPC (Agrisera, AS09 458), RbcL (Agrisera, AS03 037), Cyt f (Agrisera, AS08 306)
Validation	PetC, AtpB, NdhH, PsaB, Lhca1, Lhcb2, PsbS, PEPC and cytf have confirmed reactivity in Zea mays that is phylogenetically close to Setaria viridis (https://www.agrisera.com), RbcL and D1 have predicted reactivity in all plants, no exclusions reported so far (https://www.agrisera.com)
